# The Existence and Localization of Nuclear snoRNAs in *Arabidopsis thaliana* Revisited

**DOI:** 10.3390/plants9081016

**Published:** 2020-08-12

**Authors:** Deniz Streit, Thiruvenkadam Shanmugam, Asen Garbelyanski, Stefan Simm, Enrico Schleiff

**Affiliations:** 1 Department of Biosciences, Molecular Cell Biology of Plants, Goethe University, D-60438 Frankfurt am Main, Germany; D.Streit@bio.uni-frankfurt.de (D.S.); shanmugam@em.uni-frankfurt.de (T.S.); asen.garbelyanski@stud.uni-frankfurt.de (A.G.); stefan.simm@uni-greifswald.de (S.S); 2 Institute of Bioinformatics, University Medicine Greifswald, D-17475 Greifswald, Germany; 3 Frankfurt Institute of Advanced Studies (FIAS), D-60438 Frankfurt am Main, Germany

**Keywords:** snoRNAs, *A. thaliana*, cell fractionation, NGS, tissue specificity

## Abstract

Ribosome biogenesis is one cell function-defining process. It depends on efficient transcription of rDNAs in the nucleolus as well as on the cytosolic synthesis of ribosomal proteins. For newly transcribed rRNA modification and ribosomal protein assembly, so-called small nucleolar RNAs (snoRNAs) and ribosome biogenesis factors (RBFs) are required. For both, an inventory was established for model systems like yeast and humans. For plants, many assignments are based on predictions. Here, RNA deep sequencing after nuclei enrichment was combined with single molecule species detection by northern blot and in vivo fluorescence in situ hybridization (FISH)-based localization studies. In addition, the occurrence and abundance of selected snoRNAs in different tissues were determined. These approaches confirm the presence of most of the database-deposited snoRNAs in cell cultures, but some of them are localized in the cytosol rather than in the nucleus. Further, for the explored snoRNA examples, differences in their abundance in different tissues were observed, suggesting a tissue-specific function of some snoRNAs. Thus, based on prediction and experimental confirmation, many plant snoRNAs can be proposed, while it cannot be excluded that some of the proposed snoRNAs perform alternative functions than are involved in rRNA modification.

## 1. Introduction

Ribosome biogenesis is a complex process with multifaceted steps involving rRNA synthesis, ribosomal protein expression and subsequent transport into the nucleus, rRNA modifications and folding, as well as protein assembly [1,2,3,4,5,6]. Most of the processes occur in the nucleolus, a specialized membrane-less compartment of the nucleus [7,8,9,10,11]. Consistent with the importance of functional ribosomes, many diseases are related to failures in ribosomal assembly [12,13,14,15], and therefore, cellular mechanisms for quality control of ribosome assembly have evolved [16,17,18]. Ribosome biogenesis is regulated by many ribosome biogenesis factors (RBFs [1,2,3,4,5,6]) and small nucleolar RNAs (snoRNAs), usually components of ribonucleolar particles named snoRNPs [19].

snoRNAs are essential small RNA molecules that have been identified to regulate and guide the posttranscriptional chemical modifications of rRNA, namely 2′O-methylation and pseudouridylation [20,21,22]. They are found in many organisms including archaea (despite the absence of a nucleolus) and eukaryotes but are notably absent in bacteria. Remarkably, snoRNA are even found to be coded by the Epstein–Barr viral genome [23]. The snoRNAs are classified according to globally conserved canonical sequence motifs known as C/D box and H/ACA boxes [20,24,25].

The sequences of the C (RUGAUGA) and D (CUGA) boxes as well as the H (ANANNA) and ACA box are used to differentiate between the two main snoRNA classes [24,25]. Additionally, C/D box snoRNAs often contain the less conserved boxes C´ and D´ [26]. Interestingly, the antisense sequence for rRNA binding is located 5 nt upstream of box D or D´ [26]. The snoRNAs of the C/D box family form a complex with the conserved proteins fibrillarin (methyltransferase), Nop58, Nop56, and Snu13, and the H/ACA box snoRNAs form a complex with the proteins Nap57 (yeast Cbf5), Nhp2, Nop10, and Gar1, forming enzymatically active snoRNP complexes [27].

The formation of snoRNPs contributes not only to the function of the particle but also to the nucleolar import, and it helps to stabilize the snoRNA [28]. Modifications guided by snoRNAs are important for regulating the function of ribosomes by stabilizing the rRNA scaffold, but it is also assumed that the existence or absence of a specific rRNA modification can lead to ribosome heterogeneity [4]. In yeast, it was shown that the loss of single modifications has no or just slight effects on growth or ribosome activity, whereas mutations for multiple modification sites caused severe effects by impairing growth and by reducing translational rate [29]. In humans, the depletion of two pseudouridylation sites within 28S caused by a point mutation within DKC1 lead to dyskeratosis congenital, an inherited disease that causes abnormal skin manifestations [30,31]. In plants, the mutation of a C/D box HIDDEN TREASURE 2 (*HID2*) snoRNA leads to developmental leaf polarity and overall slow-growth defects [25] similar to the array of core ribosomal protein or ribosome biogenesis factor mutants [1,3,32,33].

SnoRNAs are discussed to be largely localized in the nucleolus and to function in rRNA modification, but recent studies expanded this functional repertoire. It was demonstrated that snoRNAs dually act in the regulation of rRNA modification and in pre-mRNA splicing as exemplified for SNORD27 in HeLa cells [34]. Moreover, a subset of snoRNAs relocated into the cytoplasm upon induction of oxidative stress, leading to the proposal by the authors that snoRNAs are involved in cytosolic stress response [35]. In humans, the snoRNAs are actively exported by nuclear export factor 3 (NEF3) [36]. Moreover, a small subset of snoRNAs is even known to be involved in rRNA processing like U3, snR30/U17, U14, and U8 [4,37].

In plants, U3, U14, and U49 were identified early based on similarity to the snoRNAs in yeast or vertebrates [38,39,40]. Here, U3 in plants was found to be transcribed by RNA polymerase III instead of polymerase II as the known form in yeast or human and to possess a different capping [38]. Moreover, on the example of U14, it was demonstrated that they are present in clusters and are transcribed in polycistronic units [39] and that the genomic region can contain both C/D box- and H/ACA box-type snoRNAs [40]. The early work on these examples led to the formulation of general principles of genomic occurrence of snoRNAs, their transcription, and maturation in plants [41]. Ever since the first plant snoRNAs were discovered, the analysis of putative snoRNAs has continued. Boosted by the published genome of *Arabidopsis thaliana* [42], the number of snoRNAs rapidly expanded based on prediction of snoRNAs and the analysis of modification sites [24,26,43]. The extension of the analysis to four different tissues led to identification of 39 novel H/ACA snoRNAs [44]. However, despite the prediction and partial confirmation of existence, not much is known about snoRNA function in plants. Only the function of the C/D box-type snoRNA HIDDEN TREASURE 2 (HID2) was explored in more detail. This snoRNA associates with 45S pre-rRNA and was shown to promote accuracy and efficiency of rRNA processing [25]. However, *HID2* mutants displayed no change in 2´-ribose methylation at position G2620, leading to the conclusion that other snoRNAs might fulfill this action as backup partners.

The global studies of the snoRNA content in *A. thaliana* were complemented by the analysis of the RNA family in rice [45,46]. This has led to the extension of predicted snoRNAome by optimizing the search algorithms [47]. Analyzing the predicted and confirmed plant snoRNAs, it was demonstrated that snoRNAs are largely conserved in the eukaryotic kingdom, while intragenomic mobility and species-specific variations of content and specificity contribute to a certain diversity in genomic organization and function [48,49].

Recently, a link between snoRNA and miRNA function has been suggested based on the identification of dicistronic genes encoding precursors processed to snoRNA and miRNA molecules [50]. Moreover, it has been demonstrated that snoRNAs are a source for the generation of another class of small RNAs, sdRNAs (snoRNA-derived small RNAs), known to be involved in the regulation of stress response [51,52,53,54] and that they occur in tandem with genes coding for class I small heat shock proteins [55]. Considering the importance of snoRNAs for pre-rRNA processing and the fact that, for many predicted modification sites, no snoRNA could be found so far, we aimed in this study for the identification of new snoRNAs. For this purpose, we studied the distribution of snoRNAs by combining deep sequencing after nuclei enrichment and computational prediction.

## 2. Results

### 2.1. Analysis of Small RNAs in Total Cell and Nuclear Lysates 

**Figure 1:**
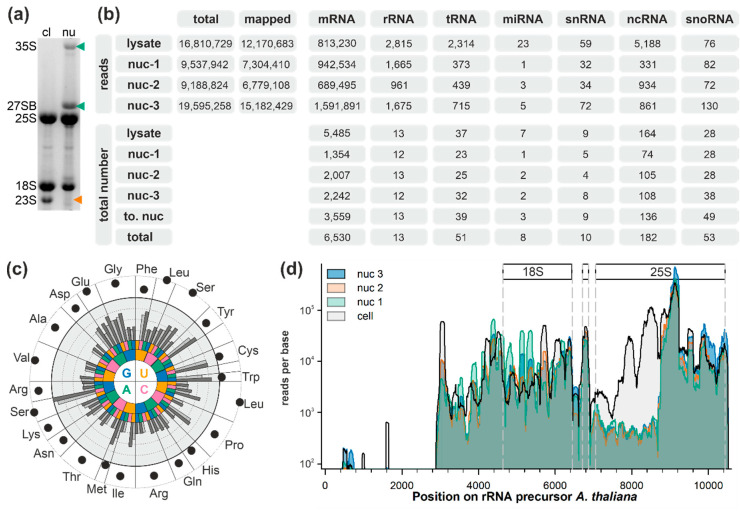
Analysis of the small RNA distribution in *Arabidopsis thaliana* cells: (**a**) RNA was isolated from cell lysates (cl) and nuclear fractions (nu) and subjected to agarose gel analysis followed by ethidium bromide staining, and migration of the rRNA precursor is indicated on the left. The presence of the 35S as well as of 27SB rRNA and the absence of 23S rRNA in the nuclear fraction are highlighted by arrowheads. (**b**) The number of reads (top) and of detected molecules (bottom) is presented. The number of total (column 2) and mapped reads (column 3) is given for each fraction. Subsequently, the number of reads mapped to genes coding for specific RNAs (according to TAIR10) is presented for cell lysate (lysate) and the three replicas of the nuclear fraction (nuc-1 to nuc-3). These ncRNAs are not annotated in TAIR10. On the bottom, the total number of identified RNAs in cell lysate (lysate), the three biological replicas of the nucleus (nuc-1 to nuc-3), accumulated results for nuclear fractions (to. nuc), or all fractions (total) are shown. (**c**) The codon is indicated as a color code from 5′ (center) to 3′ (outer rim). The total reads per base for the tRNA for each codon is shown (middle light grey background; grey bar: maximal value in one of the nuclear fractions; dark gray: cell lysate; logarithmic scale from 1 to 104). The amino acid occurrence in the proteome of *A. thaliana* is shown in logarithmic scale between 105 and 106 (outer white rim, black dot). (**d**) The reads per base found for the rRNA transcript is shown for the four experiments according to the color code on the left. The regions coding for 18S, 5.8S, and 25S are indicated.

*A. thaliana* cell suspension culture was fractionated into the nuclear depleted cytoplasm and nucleus. Ethidium bromide staining revealed two dominant pre-rRNA precursors, 35S and 27SB, in the nuclear fraction (Figure 1a, lane 2; green arrow). The specificity of these bands was verified by northern hybridization using precursor-specific probes (data not shown; probes described in [56,57]). The presence of the organellar rRNA (23S) in the cytoplasmic but not in the nuclear fraction further shows the purity of the latter (Figure 1a, lane 1; orange arrow).

The RNA from one total cell extract and from three independent nuclear isolations (nuc1, nuc2, and nuc3) was subjected to denaturing gels. RNA molecules smaller than 200 nt were excised and used for next generation sequencing. In total, 12 million reads obtained by sequencing the RNA of the total fraction as well as 7 or 15 million reads of the nuclear fractions were mapped to the genome of *A. thaliana* (Figure 1b). The total number of mRNAs identified is higher in total cell lysates when compared to the nuclear fraction (Figure 1b), although more reads were mapped to the protein coding regions. In turn, the number of identified tRNAs and rRNAs, and ncRNAs annotated in the *Arabidopsis* Information Resource (version 10; TAIR10 [58]) was rather comparable between the total cell lysate and nucleus, although the reads obtained are higher in the cell lysate (Figure 1b). Remarkably, a large number of ncRNAs were not functionally annotated in TAIR10. The number of identified snoRNAs annotated in TAIR10 was higher in the nuclear fractions than in total cell extract (Figure 1b).

For tRNAs, it has been discussed that their copy number correlates with amino acid occurrence in the proteome [59]. In here, the identified tRNAs in the total cell extract did not show a correlation with amino acid occurrence (Figure 1c). In general, the abundance of the tRNA is higher in the total cell extract (dark grey bar) when compared with the nuclear fractions (grey bar). The highest abundance was observed for tRNA priming with the AGC codon for serine. Serine together with leucine, glutamic acid, and valine are the most abundant amino acids in *A. thaliana* proteins. However, in contrast to tRNAs for serine, tRNAs for leucine integration are only medium abundant and tRNAs for valine are among the tRNAs with lowest abundance. In turn, while tryptophan and cysteine are low-abundance amino acids, a high tRNA content was identified for both. Thus, while the gene copy number appears to correlate with the amino acid abundance, a correlation between tRNA abundance and amino acid occurrence could not be observed. Considering that the theoretical amino acid content based on and the amino acid occurrence in all annotated proteins correlates with the determined amino acid content in a cell [60], this observation might suggest that tRNA expression levels play additional roles. This can be regulation of the speed of protein synthesis as established for bacteria [61] or regulation of mRNA usage for translation as established for mammals [62,63].

The general coverage of the rRNA was comparable for the three nuclear samples, while the coverage is not homogenous throughout the rRNA transcript (Figure 1d). We assume that the observed pattern of detection short regions of the rRNA with alternating read coverage (Figure 1d) is a result of the used rRNA depletion protocol before sequencing. This protocol depends on oligoprobes that are destined to cover certain regions but not the entirety of rRNAs.

A large number of reads was obtained for the isolated small RNAs mapped to the 5′ external transcribed spacer (ETS) and the mature 18S rRNA. A certain variation of reads mapping to the 5′ and the central region (bp 5600–5800) of the 18S rRNA was observed, for which a higher read coverage was observed in the nuclear or cell lysate fraction. In contrast, a large number of reads observed for the isolated RNAs from the total lysate fraction were mapped to the 5′ region of the 25S rRNA, while reads for this region were less frequent when the RNA of the nuclear fractions was analyzed. Considering the two facts that RNAs migrating at sizes smaller than 200 nt were excised from the gel and that rRNA depletion was performed, reads covering the rRNA originated from specific or unspecific rRNA breakdown. Thus, the observed difference of the coverage of the 5′ region of 25S could result from a higher stability of this rRNA in pre-ribosomal complexes dominating the rRNA pool in the nucleus when compared to mature ribosomes with high abundance in the cytoplasm. Alternatively, the analysis of *HID2* mutants uncovered a pre-rRNA degradation mode in the nucleus, degrading the 3′ end of the 27SB pre-rRNA [25]. In parallel, it has been discovered that endoribonucleases are involved in the degradation of rRNA during ribophagy [64,65], which produces small fragments of the entire 25S rRNA.

### 2.2. The snRNA and snoRNA Content in the Nucleus

**Figure 2:**
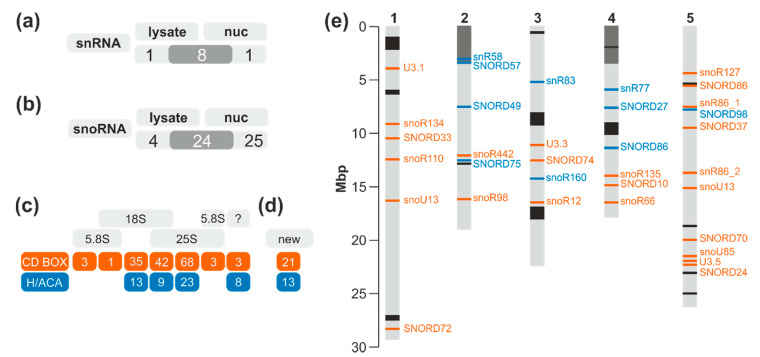
The detected snRNAs and snoRNAs in *A. thaliana* cells. (**a**,**b**) The distribution of the snRNAs (**a**) and snoRNAs (**b**) annotated in TAIR10 in cell lysate and nuclear fractions is shown. (**c**,**d**) The number of identified snoRNAs with C/D box, H/ACA, or unknown fold known present in snOPY or snoRNA DB (**c**) or additionally identified (**d**) is presented. For the annotated snoRNAs (Cc), the number for the (predicted) target rRNA is given. (**e**) The general chromosomal localization of the newly identified putative snoRNAs (**d**) is shown. The color code indicates the distribution in the two structural groups as in (**c**). rDNA (grey) and regions with high frequency snoRNAs occurrence (black; [26]) are highlighted.

The identification of snRNAs was largely comparable between nuclear fractions and the total lysate (Figure 2a). Further, almost all TAIR10 annotated snoRNAs found in the cellular extract are also found in the nuclear fraction, but additional snoRNAs are found in the nuclear fraction as well (Figure 2b). However, by in silico studies, more than 300 snoRNAs in *A. thaliana* or *Oryza sativa* [47,52] have been identified. Thus, the presence of the 223 annotated snoRNAs from the snOPY (snoRNA Orthological Gene Database) [66] and the plant snoRNA DataBase [67] was analyzed in addition to the snoRNAs annotated in the TAIR10 genome. Almost all previously predicted or identified snoRNAs (in total 208: 155 C/D box snoRNAs, 53 H/ACA snoRNAs) were identified in at least one of the three nuclear fractions (Figure 2c; Table S1) and only 15 snoRNAs deposited in snOPY or snoRNA DB were not identified by our next generation sequencing approach (Table S2).

The longest read frame covered by reads for small RNAs found in the nucleus was used to assign them to known snoRNAs based on RFAM [68]. This strategy yielded the identification of 34 snoRNAs with C/D box (21) or H/ACA motif (13) that are not yet deposited in TAIR10, snOPY, or snoRNA DB (Figure 2d; Table S3). The newly identified putative snoRNAs do not generally cluster in specific chromosomal regions (Figure 2e). Most of them were identified with high significance. However, for some RNAs, different short regions were identified that show similarity to different snoRNAs but with low significance (e.g., for snoR135; Table S3). Here, only the annotation with the lowest *p*-value is considered. In general, we followed the RFAM-based classification with one exception: the U3-like snoRNAs. While prediction suggested an H/ACA-type snoRNA, this family is generally described as C/D box snoRNA. In addition, for the newly found snoRNAs, it was searched for complementary sites on the rRNA (Table S3). In total, for 16 of the 34, such a site could be identified.

### 2.3. Localization of the U3 snoRNAs of the C/D Box Family

**Figure 3:**
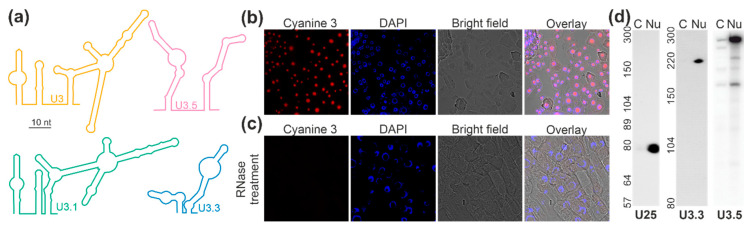
U3-like snoRNAs of the C/D box family: (**a**) The structure of the U3 snoRNAs were predicted (see the Materials and Methods section). For comparison, the structure of U3 from yeast [69] is shown. (**b**,**c**) A FISH probe (left, Table S4) against the U3-like snoRNA family was used for incubation of root cells of *A. thaliana* plants before (**b**) and after RNase treatment (**c**). DNA was visualized by DAPI staining (second), and the cell shape by recording the bright field image (third). The overlay of all signals is shown for representative cells (right). (**d**) RNA isolated from nuclear depleted cytoplasm (cy) and nuclear fraction (nu) were subjected to Polyacrylamide Gel Electrophoresis (PAGE)-based separation. The migration of the indicated U25- and the U3-type snoRNAs was probed by northern blotting with specific probes (Table S5). Migration of RNA standards is shown on the left.

The inspection of the sequence of the U3-type snoRNAs resulted in the identification of typical elements of a C/D box snoRNA and of a predicted structure comparable to U3 from yeast (Figure 3a [69]). U3 encoded by chromosome 1 (U3.1) is the plant U3 snoRNA of which the predicted structure is most comparable to the one proposed for yeast U3 (Figure 3a). The other two plant U3-like snoRNAs contain all important elements, but the predicted structure shows a certain difference when compared to the one of yeast U3 (Figure 3a). Interestingly, while U3.1 is hardly detectable, U3.3 is only lowly abundant and U3.5 is highly abundant in all fractions. The enrichment of U3.1/U3.3/U3.5 is 1:10^2^:10^6^ (Table S3).

After detection of the putative U3-type C/D box snoRNAs, the intracellular distribution was experimentally confirmed. The distribution of the three forms of U3 was probed together because U3-1, U3-3, and U3-5 are not distinguishable by a FISH probe. Using this probe for the detection of this particular snoRNA in roots of *A. thaliana*, a nucleolar distribution was observed as judged from the overlay of a FISH signal and DAPI (4′,6-diamidino-2-phenylindole) staining (Figure 3b). However, it is likely that the signal is dominated by U3.5 because this putative snoRNA shows the highest abundance when compared to the other two. The specificity of the detection was confirmed by RNase treatment of the cells before incubation with the probe (Figure 3c).

The analysis by FISH was complemented by northern blotting of cell lysate and nuclear fractions with probes against selected snoRNAs. The separation of the two fractions was confirmed by northern blot analysis of the U25 localization (Figure 3d, Nu, U25). The analysis of the U3-like snoRNAs with probes specific for different U3 forms confirmed the presence of U3-like snoRNAs in the nucleus (Figure 3d, Nu, U3.3, U3.5). While U3.1 was not detectable by northern hybridization (data not shown), U3.3 (approximately 220 nt) was only detectable in the nuclear fraction and U3.5 was predominant in nuclear fraction but in part detected in the cytoplasm as well. At least four fragments in addition to the most abundant snoRNA with approximately 300 nt were detected by northern probing of U3.5. Four of these five detected RNAs were present in the cytoplasmic fraction, but only the RNA with approximately 260 nt was enriched in the cytoplasmic fraction when compared to the nuclear fraction. In turn, the largest, the third (approximately 220 nt) and the smallest RNA (approximately 160 nt) are nuclear specific, as the predominant majority was detected in the nuclear fraction. Remarkably, the RNA consistent with the predicted length of U3.5 (219 nt) appeared exclusively in the nuclear fraction. Overall, the existence and nucleolar localization of at least two snoRNAs could be confirmed.

### 2.4. Localization of snoRNAs of the C/D Box Family

**Figure 4:**
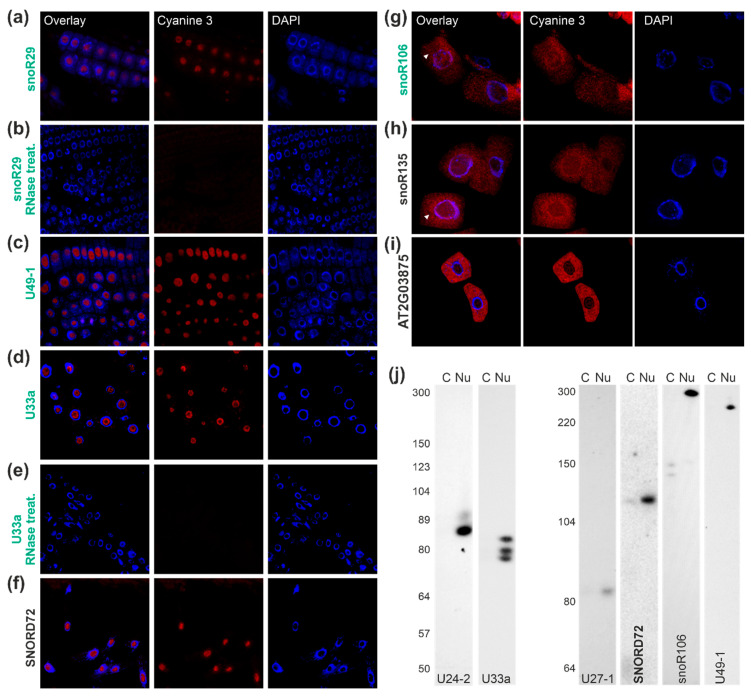
snoRNAs of the C/D box family: (**a**–**i**) FISH probes (Table S4) against C/D box snoRNAs (Tables S1–S3) were incubated with root cells of *A. thaliana* plants (second), and DNA was visualized by DAPI staining (third). The overlay between FISH probe signal and DAPI staining is shown for representative cells (first). In (**b**,**e**), images for FISH analysis after RNase treatment of the cells are shown exemplarily for snoR29 and U33a. (**j**) RNA isolated from nuclear depleted cytoplasm (cy) and nuclear fraction (nu) were subjected to acrylamide gel-based separation. The migration of the indicated snoRNAs was probed by northern blotting with specific probes (Table S5). Migration of RNA standards is shown on the left.

The intracellular distribution of additional putative C/D box snoRNAs was experimentally approached. At first, the distribution of the known C/D box snoR29 was analyzed, of which two forms exist, namely snoR29-1 and snoR29-2. For the two, we found an enrichment of 4 ± 2-fold in the nuclear fraction based on the next-generation sequencing results (Table S1). Designing a probe that targets both snoR29 variants, we observed a nucleolar distribution of snoR29 by FISH experiments on roots of *A. thaliana* as judged from the overlay of FISH signal and DAPI staining (Figure 4a). The specificity of the detection of the probe was confirmed by RNase treatment (Figure 4b).

For U49-1, a ratio between nuclear and lysate fraction of 1.6 ± 0.7 was observed (Table S1), which suggests an equal occurrence in both fractions. FISH analysis yielded a nucleolar localization (Figure 4c). To confirm the localization determined by FISH, which somewhat stands in contrast to the almost equal distribution in the fractions analyzed by NGS, the presence of the snoRNA in the different fractions was analyzed by northern blotting (Figure 4j, right). The latter yielded an exclusive detection in the nuclear fraction as well. Thus, a nucleolar localization can be concluded for U49-1. The same holds true for U33a, which is highly abundant and about 3 ± 1-fold enriched in the nuclear fraction (Table S3). The localization of U33a in the nucleolus was confirmed by FISH (Figure 4d,e) and northern blotting (Figure 4j, second). For U33a, as many snoRNAs, more than one allelic variance exists due to gene duplications [67], which are indistinguishable by a northern probe. In the databases, at least two variants of 81 nt and 82 nt are annotated [67]. Upon northern hybridization, we detected in total three fragments, which suggests that a yet unknown third variant exists (Figure 4j). Similarly, for U24, two variants are deposited in the database [67]. The probe designed for U24-2 (83 nt) partly binds to U24-1 (94 nt) as well (Figure 4j, first). In turn, the probe against U27-1 detected only one RNA, and this was found to be present in the nuclear fraction as expected (Figure 4j).

The newly discovered C/D box snoRNA SNORD72 represents a snoRNA with similar intermediate abundance in both the nuclear and the total cell lysate fraction as judged from NGS results (Table S3). FISH analysis yielded an exclusive localization in the nucleolus (Figure 4f). The analysis of the distribution by northern blotting revealed that the majority of this putative snoRNA was present in the nuclear fraction while only a small portion was detectable in the cytosolic (nuclear depleted) fraction as well (Figure 4j, fourth). This suggests that SNORD72 is mainly localized in the nucleolus as found for the above described known snoRNAs, but a small fraction is present in the cytosol as well.

For the previously assigned snoR106, a 4.3 ± 0.5-fold enrichment in the lysate fraction was observed (Table S1). FISH analysis yielded a nuclear localization, but a cytosolic signal was detected as well (Figure 4g, white triangle). However, the distribution of snoR106 was not exclusively cytosolic as determined for AT2G03875, which was used to confirm the specificity of the approach (Figure 4i). The northern blot analysis with a probe for snoR106 yielded exclusive detection of the 300 nt RNA in the nuclear fraction, while two shorter RNAs (approximately 150 nt) were detected in the nuclear depleted fraction (Figure 4j, fourth). While the enrichment cannot be directly quantified due to dissimilar fragment sizes between fractions, this observation suggests a nuclear localization of at least the larger RNA detected with the probe against snoR106.

The newly discovered snoR135 contains short regions with similarity to different snoRNAs and was low abundant and 9 ± 3-fold enriched in the total cell lysate (Table S3). In agreement with this observation, FISH analysis yielded a localization of snoR135 in the cytoplasm as well as in the nucleoplasm (Figure 4h). Therefore, this small RNA is distributed between both compartments.

### 2.5. Localization of Two snoRNAs of the H/ACA Box Family

**Figure 5:**
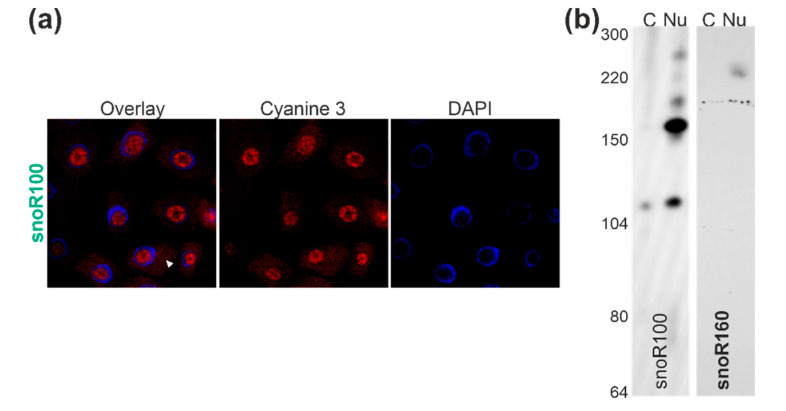
snoR100 and snoR160 of the H/ACA family: (**a**) FISH probes (Table S4) against snoR100 were incubated with root cells of *A. thaliana* plants and DNA was visualized by DAPI staining. Shown is the overlay between FISH probe signal and DAPI staining, the FISH signal, and the DAPI staining. The arrow points to cytosolic signal. (**b**) RNA isolated from nuclear depleted (cy) and nuclear fraction (nu) of *A. thaliana* cell suspension culture was subjected to acrylamide gel-based separation, and migration of indicated snoRNAs was probed by northern blotting (Table S5). Migration of nucleotide standards is shown on the left.

The number of identified H/ACA snoRNAs is lower than the number of C/D box snoRNAs (Figure 2). Nevertheless, we selected two candidates to test for their cellular localization. On the one hand, snoR100 is enriched by 2.2 ± 0.9-fold in the cell lysate fraction when compared to the nuclear fraction (Table S1); on the other hand, for snoR160, an equal abundance (1.0 ± 0.5) was observed (Table S3). FISH analysis of snoR100 yielded a nucleolar localization, but a cytosolic signal was detected as well (Figure 5a, white triangle). Northern blot analysis yielded two abundant populations for snoR100: one of about 160 nucleotides and one of about 100 nucleotides. The latter was indeed detectable in both the nuclear and the nuclear depleted cytosolic fraction (Figure 5b, left). Hence, this snoRNA might be further processed and the smaller version can be exported to the cytoplasm. In turn, for snoR160, we did not detect any cytosolic signal (Figure 5b, right).

### 2.6. Tissue-Specific Localization of Selected snoRNAs

**Figure 6:**
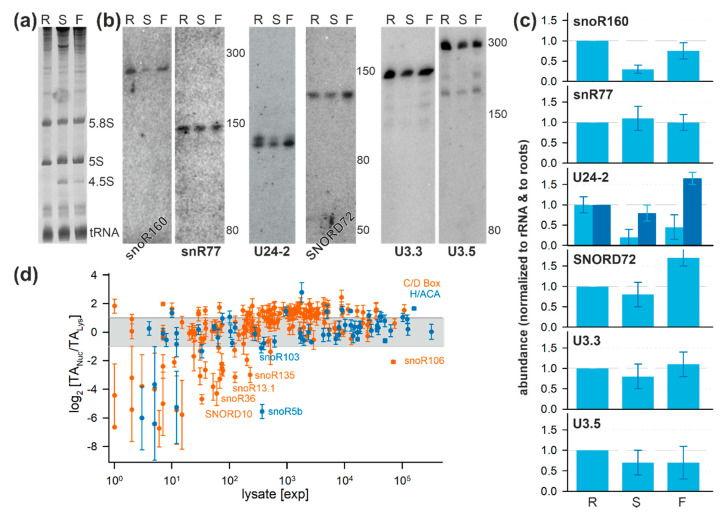
Tissue-specific distribution and localization of snoRNAs: (**a**) RNA was isolated from roots (R), shoots (S), or flowers (F) of *A. thaliana*. Ethidium bromide-staining confirmed RNA quality and was used as loading control. (**b**) Northern blot analysis of the fractions shown in (A) with probes for indicated snoRNAs: Migration of nucleotide standards is shown (left). (**c**) Density of bands (Figure 6b) was quantified and normalized to 5.8S and 5S density (Figure 6a) and to the abundance in roots. For U24-2, values for the upper (light blue) and lower band (dark blue) were normalized to the density of the lower band. (**d**) Abundance of all identified snoRNAs expressed as transcripts per million in cell lysate was plotted against the log_2_ of the ratio of the average abundance in the nucleus and lysate (SD: error bar). The color code is indicated on the right. The snoRNAs with high abundance in the lysate are named.

To determine possible tissue-specific variations of snoRNAs, RNA was isolated from roots, shoots, and flowers (Figure 6a). Probing for the low-abundance newly discovered putative H/ACA-type snoRNA snoR160 (found in cell cultures; Figure 5) and putative C/D box snoRNA snR77 (not detected in cell cultures; Table S3) yielded a signal for both (Figure 6b). Quantification of the intensities and normalization to the ethidium bromide stained small rRNA abundance (Figure 6a) suggests that snoR160 is more abundant in roots and less in shoots when compared to flowers (Figure 6c) while snR77 is equally abundant in all three tissues (Figure 6b, second; Figure 6c).

In cell cultures, two forms of the C/D box snoRNA U24-2 were detected, where the larger form was found to be less abundant (Figure 4). The two variants are present in all analyzed tissues (Figure 6b, third). However, in roots, the larger variant is as abundant as the smaller variant while the larger variant is almost absent in shoots (Figure 6b, third; Figure 6c). Moreover, the smaller transcript is enhanced in flowers when compared to roots (Figure 6c). Similarly, the C/D box snoRNA SNORD72 is higher enriched in flowers than in the other two tissues (Figure 6b, fourth; Figure 6c). In contrast, U3.3 and U3.5 are rather equally abundant in all tissues (Figure 6b, fifth, right; Figure 6c). Thus, some of the snoRNAs not detected in fractions of cell cultures by northern blotting are present in different tissues of *A. thaliana*. Moreover, the analysis of the limited set of snoRNAs already suggests that the snoRNA occurrence and abundance is in part a tissue-specific phenomenon.

## 3. Discussion

SnoRNA discovery in plants has primarily focused thus far on utilizing the genome sequences of plant species, thus heavily relying on prediction and thereafter validating the homology-based identified snoRNAs through experimental means. Furthermore, cellular compartment distribution has largely remained unknown for most snoRNAs. This prompted analysis of the abundance of transcripts in cells and in the nuclear fraction under normal growth conditions. In total, 176 C/D box and 66 H/ACA box snoRNAs were identified in one of the two analyzed fractions (Figure 2). Despite the identification of 197 snoRNAs which have been previously described and deposited in databases, 45 additional putative snoRNAs from so far not annotated chromosomal positions with similarity to known snoRNA families or structures were identified (Figure 2; Table S3). Notably, multiple snoRNAs of the same family were detected, yielding a total of 147 families (Figure 3; Tables S1 and S3). A similar situation was observed for plant ribosomal proteins and ribosome biogenesis factors, where multiple co-orthologues were found for the different protein families [70,71]. Thus, it is tempting to speculate that different snoRNPs might be formed containing distinct members of the same family. The differences in snoRNA abundance in the three tested tissues exemplified for a few candidates (Figure 6) might lead to the proposal that these complexes have a differential importance in the various tissue, during development, or in stress response.

The analyzed C/D box snoRNAs U24-2, U27-1, U33a, and SNORD72 are between 70 and 120 nucleotides as determined by northern blotting, which is typical for such snoRNAs [72]. The exceptions are U3.3 (approximately 220 nucleotides), U3.5 (approximately 300 nucleotides), snoR106 (approximately 300 nucleotides), and U49-1 (approximately 250 nucleotides). The larger size of the two U3-like snoRNAs is expected based on the comparison to the U3 in, e.g., yeast [69]. Noticeably, using probes for the snoR135, we could not detect a transcript in any of the fractions. This putative snoRNA might be of very low abundance, explaining the failure in detection by northern blotting, although a certain number of NGS reads were detected in the cytoplasm (Table S3) and as the transcript was detected by FISH before but not after RNase treatment (Figure 4). In turn, the H/ACA snoRNAs snoR100, snoR160, and snR77 consist of about 150–300 nucleotides (Figure 5 and Figure 6), which agree to the annotation of H/ACA snoRNAs, e.g., in humans [72].

**Figure 7:**
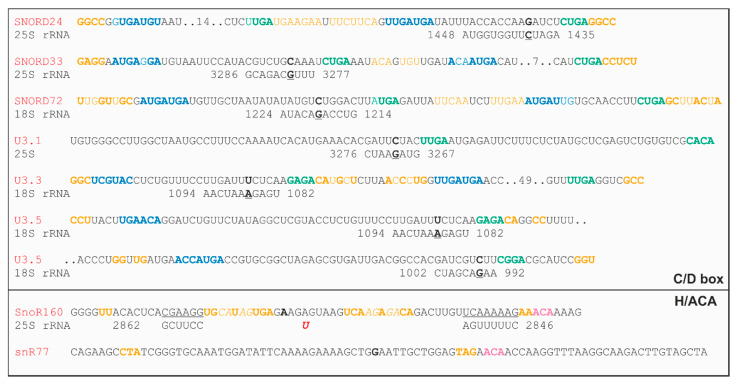
Illustration of the identification of complementary rRNA sites for newly assigned snoRNAs (Table S3): Shown are the sequences of newly assigned C/D box and H/ACA snoRNAs and the putatively modified rRNA regions. In yellow is putative duplex structures, in blue and green is C/D box elements, and in magenta, ACA are highlighted. These sites were not experimentally confirmed.

Based on prediction, for some of the newly discovered snoRNAs, a complementary rRNA sequence could be identified. For example, it can be proposed that the C/D box snoRNA SNORD72 is involved in 2′-O-methylation of G1219 in the 18S rRNA (Figure 7; Table S3). Further, U3.3 and U3.5 target the same region in 18S for modification of A1087 while U3.5 additionally targets 18S for modification of G995. In contrary, our prediction suggests that U3.1 is involved in 25S modification (Figure 7; Table S3). Interestingly, snoR160 targets a region in 25S required for converting uridine to pseudouridine at U2855. This position was just recently discovered [73]. In contrary, for some putative snoRNAs, the prediction of a complementary rRNA sequence was not possible as exemplified for snR77 (Figure 7; Table S3).

However, small RNAs with similarity to H/ACA and C/D box snoRNAs that are not involved in rRNA modification have been described in the past. One class is formed by small RNAs in Cajal bodies, so-called scaRNAs, which are discussed to be involved in the modification of spliceosomal small nuclear RNAs [34,74] or tRNAs [75]. Thus, some of the snoRNAs assigned might rather belong to this family. In addition, some of the snoRNAs identified might be a fragment of a larger transcript, as long noncoding RNAs that contain 5′ and 3′ ends shaped like a snoRNA (sno-lncRNAs) have been previously discovered [76]. Thus, some of the 15 previously assigned snoRNAs not detected here might represent such ends, while others might simply be expressed in a tissue or developmental stage-specific manner. Moreover, it is worth mentioning that only small RNA molecules were isolated for our analysis, and as exemplified by the detection of mRNA and rRNA fragments (Figure 1), some of the snoRNAs assigned here might be part of larger transcripts as well.

Some of the previously or here assigned snoRNAs were found to be enriched in the cell lysate fraction but are not equally abundant or enriched in the nuclear fraction (Figure 6d). On the one hand, for some of the snoRNAs this observation might result from the low abundance and, hence, a low significance of detection (Figure 6d, >50 transcripts per million). On the other hand, for others, the enrichment in the cell lysate is rather high. It has been proposed that cytosolic distribution of snoRNAs might be a regulatory circuit, e.g., involved in the stress response [35,36]. Consistent with this notion, snoRNAs and snoRNA-derived small RNAs (sdRNA) have been identified in cytoplasmic and ribosomal fractions upon stress treatment in yeast [77,78]. It was discussed that such snoRNAs and sdRNAs regulate the translation activity of ribosomes during stress-induced reprogramming of the proteome. However, the activity of such cytoplasmic snoRNAs and sdRNAs in plants needs to be elucidated in the future.

Furthermore, for snoR100, a 120-nucleotide long transcript was detected in addition to the full length as expected from prediction, which is partially present in the nuclear depleted cellular fraction (Figure 5). Similarly, smaller fragments that are in part or entirely localized in the cytosol were observed for snoR106 and U3.5 (Figure 3 and Figure 4). These smaller fragments might represent RNAs that are derived from snoRNAs known to play a regulatory role as previously discussed [65] but not yet systematically explored in plants. Thus, two complementary or alternative hypotheses can be formulated for the occurrence of snoRNAs or snoRNA fragments in the cytosol.

In summary, by RNA sequencing after cell fractionation, we confirmed the existence of most of the previously assigned snoRNAs in the nuclear fraction. In addition, some yet unexplored snoRNAs, like the three members of the U3 family, were discovered. Moreover, our analysis points to a cytosolic localization of snoRNAs or sdRNA in plants. Therefore, plant snoRNAs and sdRNA likely have regulatory functions in addition to the contribution to ribosome biogenesis as established for other organisms [79]. In addition, we provide experimental evidence for a tissue-specific profile of some of the snoRNAs analyzed. Consequently, our findings raise the question concerning the functions of a large number of snoRNA in plants in different tissues, cellular compartments, and molecular complexes.

## 4. Materials and Methods

*Cell Culture and plant growth*—*A. thaliana* cell culture was grown and maintained as described [80]. *A. thaliana* cultivation on soil and plates was performed as previously described in detail [56].

RNA isolation—RNA isolation from different plant tissues was performed as established [56,57]. For root samples, root tissues from 30-day-old plants grown on Murashige and Skoog media were collected and processed after removing the adhered media with tissue towels. For shoot samples, aboveground shoot tissues of 20-day-old plants were used from soil grown pots. For next generation sequencing, RNA was purified [57] from total cell extract and nuclear extract (nuc-1, nuc-2, and nuc-3) prepared as described [81]. Nuclear-free cytoplasmic extract RNA for northern hybridization was prepared as follows: 10 mL cell culture were filtered, grounded using liquid nitrogen, and resuspended in 3 mL of HNB buffer (5% sucrose, 5% glycerol, 25 mM HEPES pH 7.5, 25 mM NaCl, 5 mM MgCl_2_, 1 mM EDTA pH 8.0, 2 mM CaCl_2_, and 10 µL/mL Protease Inhibitor Cocktail; P9599, Sigma). After 15 min incubation on ice, NP-40 was added (1% final concentration), followed by thorough mixing; 3 mL of homogenate was loaded onto 1 mL of 10% sucrose in HNB buffer and centrifuged at 4 °C at 2.150 *g* for 10 min. The supernatant consisted of cytoplasmic extract, which was used for RNA purification [56,57].

*RNA sequencing*—RNA libraries of total cell extract and nucleus were size separated on polyacrylamide gels. RNAs smaller than 200 nucleotides were extracted. RNA was prepared for next generation sequencing by GenXPro (Frankfurt, Germany), including rRNA depletion. Stranded single-end reads of 100 nucleotides were created on Ilumina NextSeq 500 and controlled for their quality by FASTQC (www.bioinformatics.babraham.ac.uk/projects/fastqc). Reads were mapped to the genome of *A. thaliana* (TAIR10 [58]) with NextGenMap [82] using standard parameter settings. Genomic regions with mapped reads were analyzed using HTSeq [83] for each RNA-type individually.

*RNA analysis*—The analysis of known snoRNAs in plants was performed based on the annotated plant snoRNAs deposited in snOPY [66] and snoRNA DB [67] (Table S1; Table S2). tRNA (plantRNA [84]) and rRNA sequences (deposited in SILVA [85] or published [86]) were used to assign reads to these RNA classes. The different databases served as reference for mapping with NextGenMap. Values for mapped reads (Table S1; Table S3; Figure 6) are presented as transcripts per million reads.

*Detection of new snoRNAs in A. thaliana*—Detection of new snoRNAs (Table S3) in the nucleus was based on a filtered set of reads from nuclear samples cleaned from all reads mapped to annotated “Genes” in the GFF file of *A. thaliana* (TAIR10 [58]). Regions of continuous read coverage in all three samples were treated as “contigs” of putative ncRNAs. These “contigs” were used as input for Infernal [87] to identify similarity based on the sequence and structure prediction in the covariance models to RNAs deposited in RFAM [68]. “Contigs” with similarity to a snoRNA were extracted and classified as box C/D or H/ACA snoRNAs. Possible rRNA modification sites were predicted with Snoscan [88] and RNAsnoop [89].

*Fluorescence in situ hybridization (FISH)*—RNA-FISH was performed in *A. thaliana* roots as described [90]. The snoRNA binding anti-sense oligos for FISH (Table S4) were labeled with cyanine-3 on 5′ and 3′ ends (Sigma-Aldrich, Darmstadt, Germany). Briefly, 1 cm of root tip from 4–5-day-old seedlings were cut and placed onto a glass dish containing 4% paraformaldehyde and fixed (30 min, room temperature (RT)). The fixative was removed, and root tips were washed with 175 mM NaCl, 1.86 mM NaH_2_PO_4_, and 8.4 mM Na_2_HPO_4_). The root tips were arranged onto a glass slide, covered, and gently squashed, and the glass slides with coverslip was immersed in liquid nitrogen for 5 s. The cover slip was removed from the squashed root cells, and tissues were air dried (30 min, RT) and subsequently immersed in 70% ethanol on a coplin jar (>1 hr, RT). Before hybridization, slides were removed and ethanol was evaporated. Roots were washed twice with wash buffer (10% formamide; 300 mM NaCl and 30 mM sodium citrate, pH 7.0). Cy-3 labeled probes were mixed (final 250 nM) in 100 µL hybridization buffer (wash buffer plus 100 mg/mL dextran sulphate; 42867 Sigma-Aldrich, Darmstadt, Germany), added to roots, and incubated in a humid chamber box (>12 h, 37 °C, dark). Cover slips were removed, and roots were washed twice with 200 µL wash buffer and incubated in wash buffer (30 min, 37 °C, dark). The nuclei were stained with 100 µL of DAPI solution (100 ng/ µL in wash buffer; 30 min, 37 °C, dark). After washing twice with 300 mM NaCl and 30 mM sodium citrate, pH 7.0, samples were incubated for 2 min with 100 µL of Anti-fade GLOX buffer (0.4% glucose, 10 nM Tris-HCl, 300 mM NaCl, and 30 mM sodium citrate, pH 7.0) followed by incubation in 100 µL of Anti-fade GLOX buffer containing enzymes (1 µL each of glucose oxidase, Sigma G0543; Catalase, Sigma C3155). A coverslip was placed and sealed with nail varnish. For imaging, Zeiss LSM780 Confocal Laser scanning microscope was used in which Cy3, and DAPI were excited at 543 nm and 405 nm respectively while the emission signal was recorded at 570 nm and 460 nm, respectively. All images were prepared using Image J [91].

*Northern blotting and hybridization*—For northern blotting, 5 µg each of cytoplasmic and nuclei RNA was separated alongside low-range ssRNA marker on 12% denaturing PAGE containing 7 M urea in TBE (90 mM Tris, 90 mM boric acid, and 2 mM EDTA pH 8.0). RNA was mixed with equal volume of 2× formamide-loading buffer (95% formamide, 0.025% bromophenol blue, 0.025% xylene cyanol FF, 0.025% sodium dodecyl sulphate, and 5 mM EDTA), heat denatured (85 °C, 5 min), and placed on ice before loading. After electrophoresis, gels were stained with ethidium bromide and electro-blotted onto a HvBond Nylon membrane (RPN303B, GE Healthcare, Buckinghamshire, UK). RNA was UV cross-linked with the membrane; 50-pmol oligonucleotides (Table S5) were 5′ end labeled with [γ-^32^P]-ATP (SRP 201, Hartmann Analytic) in a 20 µL reaction using polynucleotide kinase according to manufacturer’s instructions (EK0031 Thermo Scientific, Darmstadt, Germany) and subsequently added onto the membrane pre-hybridized with 50 mL of hybridization buffer (6X SSC, 0.1% sodium dodecyl sulphate, 2% Denhardt´s solution (2% bovine serum albumin, 2% ficoll 400, 2% polyvinylpyrrolidone) and 0.05 mg/mL MB-grade DNA (11467140001, Roche Diagnostics GmbH) and was shaken overnight at 37 °C. The membranes were washed with low and high stringency buffer containing 6X SSC and 2X SSC plus 0.1% SDS, respectively. Radioactivity was imaged using Typhoon scanner (GE Healthcare, Uppsala, Sweden) and quantified with Image J [91].

## Supplementary Materials

The following are available online at https://www.mdpi.com/2223-7747/9/8/1016/s1, Table S1. Identified known snoRNAs: Listed is the name of the snoRNA extracted from snOPY or snoRNADB; the strand-specific transcript abundance values for nuclear experiments 1, 2, and 3; as well as that for the total cell lysate. “-“ indicates the absence of a signal, and 0 indicates read counts below threshold. The next columns indicate the classification as C/D box or box H/ACA snoRNA, the targeted rRNA, the genomic organization, and the genomic localization. The final four columns show the predicted length, the chromosome, and the start and end position in the chromosome of the coding region. If alternative length of the regions were deposited in TAIR, snOPY, or snoRNA DB one representative is presented. Table S2. snoRNAs deposited in snoRNAs, snOPY, or TAIR not identified: Listed is the name of the snoRNA extracted from snOPY or snoRNA DB, the classification as C/D box or box H/ACA snoRNA, the targeted rRNA, the genomic organization, and the genomic localization. The final four columns show the predicted length, the chromosome, and the start and end position in the chromosome of the coding region. If alternative length of the regions were deposited in TAIR, snOPY, or snoRNA DB, one representative is presented. Table S3. snoRNAs with an identified new coding region: Listed is the name of the snoRNA assigned based on the best RFAM hit; the mapped region (chromosome, start and end position); the strand-specific transcript abundance value for nuclear experiments 1, 2, and 3; as well as for the total cell lysate. The next columns indicate the *p*-value for the given snoRNA in RFAM, the start and end position of the predicted snoRNA, the classification as C/D box or box H/ACA snoRNA, the predicted rRNA to be targeted, the start and then the end position within the rRNA, as well as the modification site in the rRNA. The last column gives the overlap of the identified region with a genomic region coding for other known snoRNAs. Table S4. FISH probes: Given is the name and the nucleotide sequence of the probe used for FISH analysis. Table S5. Northern probes: Given is the name and the nucleotide sequence of the probe used for northern blot analysis.
